# Design and Analysis of the IGBT Heat Dissipation Structure Based on Computational Continuum Mechanics

**DOI:** 10.3390/e22080816

**Published:** 2020-07-26

**Authors:** Xin Lin, Huawei Wu, Zhen Liu, Baosheng Ying, Congjin Ye, Yuanjin Zhang, Zhixiong Li

**Affiliations:** 1 School of Automobile and Traffic Engineering, Wuhan University of Science and Technology, Wuhan 430081, China; 201823704060@wust.edu.cn (X.L.); baoshengyang@wust.edu.cn (B.Y.); 2 Hubei Key Laboratory of Power System Design and Test for Electrical Vehicle, Hubei University of Arts and Science, Xiangyang 441053, China; liuzhen@hbuas.edu.cn (Z.L.); 11657@hbuas.edu.cn (C.Y.); 11564@hbuas.edu.cn (Y.Z.); 3 School of Automotive and Traffic Engineering, Hubei University of Arts and Science, Xiangyang 441053, China; 4 School of Mechanical, Materials, Mechatronic and Biomedical Engineering, University of Wollongong, Wollongong, NSW 2522, Australia

**Keywords:** IGBT, heat dissipation, thermal simulation, numerical simulation

## Abstract

With the trend of high integration and high power of insulated gate bipolar transistor (IGBT) components, strict requirements have been placed on the heat dissipation capabilities of the IGBT devices. On the basis of traditional rectangular fins, this paper developed two new types of heat-dissipating fins to meet the high requirements of heat dissipation for the IGBT devices. One is the rectangular radiator with a groove length of 2.5 mm and a width of 0.85 mm, the other is the arc radiator with the angle of 125 arc angle, 0.8 mm arc height, and 1.4 mm circle radius. After theoretically calculating the IGBT junction temperature, numerical simulations have been implemented to verify the theoretical result. The commercial CFD software, STAR-CCM+, was employed to simulate the heat dissipation characteristics of the IGBT module under different wind speeds, power, and fin structures. By analyzing the temperature field and vector field of the IGBT module, the analysis results demonstrate that the error between the simulation result and the theoretical calculation is within 5%, which proves the feasibility of the newly designed heat-dissipating fins. When the wind speed is 12.5 m/s, the power is 110 W, the fin height is 31.2 mm, and the fin thickness is 2.3 mm, the rectangular radiator can achieve the best heat dissipation performance.

## 1. Introduction

With the development and utilization of non-renewable energy such as oil and natural gas, its reserves are declining, which accelerates the demand for new energy vehicles. Due to the high switching frequency and harsh working conditions of the new energy vehicles on the road, the reliability and stability of the insulated gate bipolar transistor (IGBT) is highly required. As a result, it is crucial to investigate the IGBT operation characteristics [1,2,3,4,5].

IGBT is a new type of power semiconductor component that combines the high input impedance of the metal-oxide-semiconductor field-effect transistor (MOSFET) with the low on-state voltage drop of the giant transistor (GTR) [6,7]. The IGBT can be thought of as a special switch with the advantages of voltage control, low saturation pressure drop and high voltage withstand. The IGBT module generates a great deal of heat during the working process. If the heat is not conducted in time, it will burn the IGBT module. Thus, it is imperative to develop effective heat-dissipating/cooling components for IGBT [8,9].

At present, the cooling of most IGBT modules mainly adopted air cooling and liquid cooling [10]. With the rapid development of the IGBT modules, natural convection can no longer meet the heat dissipation requirements. It is necessary to use cooling methods such as forced air cooling and liquid cooling. Zhang et al. [11] studied the heat-dissipating needles of a motor controller. Their results showed that the cylindrical heat-dissipating needle can reduce the flow resistance of the coolant, and the heat dissipation performance of the IGBT module was greatly improved. Yan et al. [12] designed a new type of loop heat pipe. The analysis results showed that the heat pipe can efficiently transfer the heat of the IGBT module outside of the module, which greatly reduced the module temperature. Crawford et al. [13] made a detailed study on miniature heat pipes. The analysis results showed that the miniature heat pipe can significantly improve the effective thermal conductivity and the heat dissipation performance of the IGBT module. Compared with the liquid radiators, the structure of the air-cooled radiator is much simpler, the cost is lower, and the maintenance workload is smaller [14], it is reasonable to employ the air-cooled radiator in the IGBT modules to meet the heat dissipation requirements. In recent years, adoption of radiator fins, and in particular the fin structure design, has prevailed over the existing air-cooled radiators in the IGBT modules. However, there is still a long way to go to completely solve the IGBT heat-dissipating problem. An applicable fin structure in real practice of the IGBT modules remains a challenge.

In order to address aforementioned issue, two different types of heat dissipating fins are designed in this paper. The heat dissipation characteristics of the IGBT modules under different factors are studied. The temperature variation law of the IGBT module is obtained. The analysis results demonstrate the feasibility of the newly designed heat-dissipating fins in practical applications.

## 2. Proposed Geometry Model

**Figure 1:**
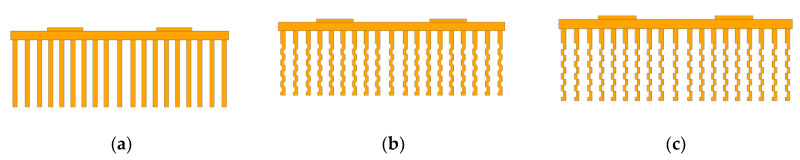
Three types of heat-dissipating fin structure. (**a**) Traditional rectangular fins; (**b**) The proposed arc-shaped fins; (**c**) The proposed rectangular fins.

A simplified model of an IGBT module is established by the three-dimensional software UG. As shown in Figure 1, the model mainly includes one aluminum substrate, four main chips and 19 copper heat-dissipating fins. The length of the substrate is 214 mm, the width is 98 mm, the height is 4 mm; the length of the main chip is 30 mm, the width is 16 mm, the height is 1.5 mm. The surface area of the heat-dissipating fin is an important factor affecting the heat dissipation of the IGBT module. Under the same conditions, the larger surface area of the heat-dissipating fin will provide better heat dissipation effect. Figure 1b,c shows the designed arc-shaped and rectangular fins in this paper. The flowability of air is another important factor affecting the heat dissipation of the IGBT module. With the same wind speed, if the distance between the fins is too small, the air flowability will be poor, and the heat dissipation effect will be bad. If the distance between the fins is too large, the air will spread to the surroundings, and the heat dissipation effect will also be deteriorated. Therefore, proper inter-flange moments are important. In order to transfer more heat from the IGBT, the distance between the heat-dissipating fins close to the main chip is set smaller, and the distance between the heat-dissipating fins away from the main chip is set larger.

Figure 1a is the traditional rectangular fins, where the fin length is 214 mm, the width is 2 mm, and the height is 30 mm. In Figure 1b the arc of the fin is 125 degrees, the arc height is 0.8 mm, and the circle radius is 1.4 mm. In Figure 1c the rectangular groove length is 2.5 mm and width is 0.85 mm.

## 3. Theoretical Thermal Calculation

The heating power of the designed IGBT model is 50 W. In the actual working process, the power loss by the freewheeling diode is very limited, so it is ignored in this study. Let us assume that all power loss is generated by the IGBT. The heat generated by the IGBT is mainly transmitted by heat conduction, first to the substrate, then to the heat sink, and finally to the air. Therefore, the thermal resistance of the entire heat transfer process of the IGBT module can be simplified to three parts. The first part is the contact thermal resistance of the IGBT chip to the heat sink, the second part is the solid thermal resistance in the heat sink, and the third part is the convective heat transfer resistance between the heat sink and the outside air [15,16,17].

The total thermal resistance of the IGBT module of the traditional rectangular fins is:

(1) $$R=R_{jc}+R_{ch}+R_{ha}$$

where:

(2) $$R_{jc}=\frac{m}{KA}$$

where *m* is the distance from the IGBT chip to the radiator, *K* = 375 $Wm^{-1}K^{-1}$ is the thermal conductivity, and *A* is the cross-sectional area between the IGBT chip and the radiator.

(3) $$\left\{ \begin{array}{l} R_{jc}=0.0374KW^{-1}\\ R_{ch}=\frac{h}{1.16K_{l}Sdn} \end{array}\right.$$

where h is the height of the radiator, $K_{l}$ = 398 $Wm^{-1}K^{-1}$ is the thermal conductivity of the radiator, *S* is the length of the heat-dissipating fin, d is the thickness of the heat-dissipating fin, and *n* is the number of heat-dissipating fins. Hence:

$$R_{ch}=0.0084KW^{-1}$$

In order to judge the specific state of the fluid in the flow field around the radiator, it is necessary to estimate the Reynolds number of the fluid. The expression of Reynolds number is as follows:

(4) $$Re=\frac{\bar{v}Y}{\gamma }$$

where $\bar{v}$ is the average velocity of air; Y is the characteristic length; γ is the kinematic viscosity of air. The physical parameters of air at a qualitative temperature of 27 °C were found. Its kinematic viscosity is $1.572\times 10^{-5}$ m^2^/s and Prandtl number is 0.702. Thus, Re≈13613.

Since the Reynolds number is greater than 4000, the fluid is in a turbulent state. Therefore, analogy can be used to analyze the convective heat transfer of the turbulent flow outside the radiator fins. Before analysis, the following assumptions need to be made:

(1) When performing forced air cooling, it is assumed that the working atmospheric pressure and relative humidity of the power device are lower than 90%.
(2) The speed of air is much smaller than the speed of sound, and the wind speed $\bar{v}$ appearing after the text is the average wind speed.
(3) The air around the radiator is in a stable turbulent state.
(4) The working environment temperature range of the radiator is −50–300 °C.

Since the air flow around the radiating fins is in a turbulent state, the correlation formula of the turbulent local friction coefficient $C_{f}$ is:

(5) $$C_{f}=0.0592Re^{-1/5}$$

where Re is the Reynolds number, which is equal to the ratio of inertial force to viscous force. The local Nusselt number can be expressed in the following form:

(6) $$Nu=\frac{\alpha_{x}Y}{\lambda }=0.332Re^{1/2}Pr^{1/3},0.6\leq Pr\leq 15$$

where $\alpha_{x}$ is the air convection heat transfer at a certain position along the length of the fin; λ is the thermal conductivity of air; Y is the characteristic length; Pr is the Prandtl number. Equation (6) can be rewritten in the following form:

(7) $$St_{x}=\frac{Nu}{RePr}=\frac{\alpha_{x}}{\rho C_{p}\bar{v}}=0.332Re^{-1/2}Pr^{-2/3}$$

where $St_{x}$ is the Stanton number, which is a criterion for describing forced convection. The larger the Stanton number, the stronger the convection heat transfer process between the fluid and the solid surface. ρ is the density of air; $C_{p}$ is the specific heat capacity of air.

According to the turbulence analogy relationship:

(8) $$St_{x}Pr^{2/3}=\frac{C_{f}}{2}$$

The expression of the local heat transfer coefficient can be obtained:

(9) $$\alpha_{x}=\frac{C_{f}\rho C_{p}\bar{v}}{2Pr^{2/3}}$$

(10) $$Re=\frac{\bar{v}x}{\gamma }$$

Substituting Formula (5) and Formula (10) into Formula (9), the local heat transfer coefficient is:

(11) $$\alpha_{x}=\frac{0.0296x^{-1/5}\rho C_{p}{\bar{v}}^{4/5}}{\gamma^{-1/5}Pr^{2/3}}$$

where *x* is the distance from a certain point to the fin. The measured data of the air properties of the radiator working environment temperature between −50 °C and 300 °C are obtained. After curve fitting the data, the function expressions of air density ρ, specific heat capacity $C_{p}$, kinematic viscosity γ and Prandtl number Pr with respect to temperature T are obtained [18] as:

(12) $$\left\{ \begin{array}{l} \rho (T)=-1.9\times 10^{-8}\times T^{3}+1.4\times 10^{-5}\times T^{2}-0.0048\times T+1.3\\ C_{p}(T)=-1.6\times 10^{-6}\times T^{3}+0.0012\times T^{2}-0.087\times T+10^{3}\\ \gamma (T)=(-4.1\times 10^{-8}\times T^{3}+0.0001\times T^{2}+0.088\times T+13)\times 10^{-6}\\ Pr(T)=-2\times 10^{-9}\times T^{3}+1.3\times 10^{-6}\times T^{2}-0.00032\times T+0.71 \end{array}\right.$$

Substituting Equation (12) into Equation (11), the average heat transfer coefficient expression is obtained as follows:

(13) $$\alpha_{x}=\frac{0.0296x^{-1/5}\rho (T)C_{p}(T){\bar{v}}^{4/5}}{\gamma {\left(T\right)}^{-1/5}Pr{\left(T\right)}^{2/3}}$$

Integrating $\alpha_{x}$ within the fin length S, the average heat transfer coefficient α can be obtained as $\alpha =\frac{1}{S}\int_{0}^{S}\alpha_{x}d_{x}$; then the average heat transfer coefficient of the air on the fin is:

(14) $$\alpha =\frac{0.037S^{-1/5}\rho (T)C_{p}(T){\bar{v}}^{4/5}}{\gamma {\left(T\right)}^{-1/5}Pr{\left(T\right)}^{2/3}}$$

Hence, the convective heat transfer resistance between the radiator and the air is:

(15) $$R_{ha}=\frac{1}{\alpha A_{r}}=\frac{\gamma {\left(T\right)}^{-1/5}Pr{\left(T\right)}^{2/3}}{0.037S^{-1/5}A_{r}\rho (T)C_{p}(T){\bar{v}}^{4/5}}$$

where $A_{r}$ is the effective heat dissipation area of the radiator. Therefore, when the ambient temperature is 26.85 °C, the convective heat transfer thermal resistance is:

$$R_{ha}=0.0667\;KW^{-1}$$

and:

$$R=R_{jc}+R_{ch}+R_{ha}=0.1125\;KW^{-1}$$

**Figure 2:**
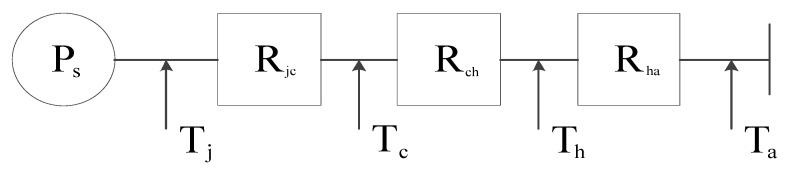
Thermal system equivalent network.

When theoretically calculating the IGBT junction temperature, the entire heat dissipation system can be simplified to the following computing network, as shown in Figure 2.

Where $P_{s}$ is the total heating power of the IGBT module; $T_{c}$ is the temperature of the substrate; $T_{h}$ is the average temperature of the radiator; $T_{a}$ (=26.85 °C) is the external ambient temperature. One can obtain that:

(16) $$T_{j}=T_{c}^{}+P_{s}\cdot R_{jc}=T_{h}+P_{s}\cdot (R_{jc}+R_{ch})=T_{a}+P_{s}\cdot (R_{jc}+R_{ch}^{}+R_{ha})$$

Thus, the junction temperature of the IGBT of the traditional rectangular fins is 49.35 °C.

Similarly, the junction temperature of the IGBT of the arc-shaped heat-dissipating fins is 48.12 °C; the junction temperature of the IGBT of the rectangular heat-dissipating fins is 47.07 °C. The theoretical temperature values of the arc-shaped and rectangular heat-dissipating fins designed are smaller than that of the traditional rectangular fins, which proves that the design is reasonable.

## 4. Numerical Simulation

### 4.1. Simulation Theory Basis

All fluid flow and heat transfer processes follow three basic conservation laws, including mass conservation, momentum conservation, and energy conservation laws. They are collectively referred to as the Navier–Stokes equation [19,20,21].

The mass conservation equation refers to the mass of the unit in the fluid that increases in unit time, which is equal to the mass of the unit body per unit time, as described in Equation (17):

(17) $$\frac{\partial \rho }{\partial t}+\frac{\partial }{\partial x_{i}}(\rho v_{i})=S_{m}$$

where ρ is the density, t is the time, and $S_{m}$ is the source term.

The momentum conservation equation refers to the rate of change of the fluid momentum of a unit in a fluid with respect to time, which is equal to the sum of all external forces received by the unit, as described in Equation (18):

(18) $$\frac{\partial }{\partial t}(\rho v_{i})+\frac{\partial }{\partial x_{j}}(\rho v_{i}\mu_{j})=-\frac{\partial p}{\partial x_{i}}+\frac{\partial \tau_{ij}}{\partial x_{j}}+\rho g_{i}+F_{i}$$

where p is static pressure; $\tau_{ij}$ is dissipative part of stress tensor; $\rho g_{i}$ is gravity; $F_{i}$ is external force.

The energy conservation equation refers to the rate of change of energy of a unit body in a fluid, which is equal to the net flow into the unit body and the work done by the physical and surface forces on the unit body, as described in Equation (19):

(19) $$\frac{\partial (\rho E)}{\partial_{t}}+\frac{\partial [v_{i}(\rho E+p)]}{\partial x_{i}}=\frac{\partial }{\partial x_{i}}(K_{eff}\frac{\partial T}{\partial x_{i}})-\sum_{\alpha }\frac{\partial }{\partial x_{i}}(h_{\alpha }J_{\alpha ,i})+\tau_{ij}\frac{\partial v_{i}}{\partial x_{j}}+S_{}$$

where $K_{eff}$ is the effective heat transfer coefficient; E is the internal energy and $h_{\alpha }$ is the enthalpy of the chemical component α; S is a source term.

### 4.2. Simulation Model

The polyhedral mesh was used to establish the simulation models (see Figure 1) in the STAR-CCM+ environment. The mesh size of the external flow field, the substrate, and the main chip was set to 1 mm. The number of boundary layers was set to three layers. The mesh size of the heat-dissipating fins was 0.1 mm. The heat dissipation method adopted forced air cooling, the air density was 1.18415 $kg\cdot m^{-3}$, the thermal conductivity was 0.02603 $Wm^{-1}K^{-1}$, and the specific heat capacity was 1003.62 $J\cdot {\left(kg\cdot k\right)}^{-1}$. In this paper, the heat sink was made of copper, the substrate was made of aluminum, and the main chip was made of silicon. The material properties of each component are shown in Table 1. A single IGBT power was set to 50 W, the air inlet boundary was set to the speed inlet with a speed of 8 m/s, and the air outlet boundary used the pressure outlet.

### 4.3. Simulation Results

Through the simulation calculation, the distribution of the temperature field and velocity vector field of the IGBT modules with arc-shaped, rectangular and traditional rectangular fins were obtained. The specific distribution from the lowest temperature to the highest temperature (A–F) of the temperature field cloud map is shown in Table 2.

**Figure 3:**
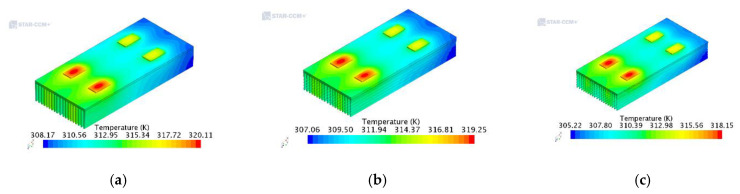
Simulation results under different heat dissipation structures. (**a**) Traditional rectangular fins; (**b**) Arc-shaped fins; (**c**) Rectangular fins.

**Figure 4:**
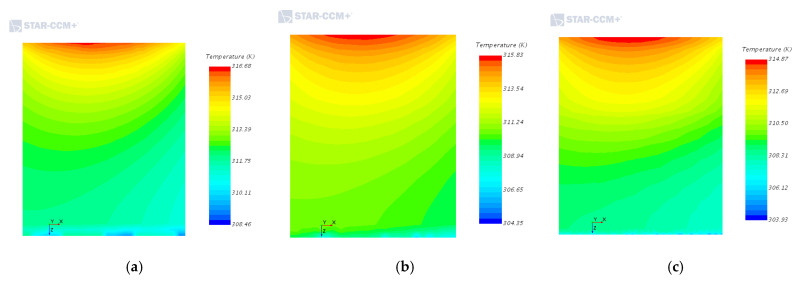
Simulation results of the cut surface of the air inlet chip of different heat dissipation structures. (**a**) Traditional rectangular fins; (**b**) Arc-shaped fins; (**c**) Rectangular fins.

Figure 3a is the temperature field cloud diagram of the traditional rectangular fins. It can be seen from the scale that the maximum temperature is 320.11 K (=46.96 °C), and the temperature gradient distribution gradually increases along the negative direction of the x-axis. But its main distribution is still around the main chip, the highest temperature is on the heat source IGBT, and the temperature difference between the inlet and outlet is about 12 K. As shown in Figure 3a, the average temperature of the IGBT in the second row along the negative direction of the x-axis is as high as 320 K. The main reason is that the front IGBT transmits most of the heat under the action of the air current. It can also be seen from Figure 4a that, at the same time as the heat generated by the chip at the air inlet of the module is transmitted downward, most of the heat is transferred to the rear of the module due to the action of the wind field, which makes the back end temperature significantly higher than the front end.

Figure 3b shows the temperature field cloud diagram of the arc-shaped fins. It can be seen from the scale that the maximum temperature is 319.25 K (=46.10 °C), the temperature value increases gradually along the negative direction of the x-axis, and the temperature is highest in the IGBT of the second row. However, the heat dissipation of the IGBT group in the front row is more obvious, mainly due to the greater influence of the wind field received here. It can be seen more clearly from Figure 4b that, compared with the traditional rectangular fins, the temperature distribution of the chip cut surface at the air inlet is more uniform, and the heat dissipating effect is remarkable. The maximum temperature at the chip is 315.83 K, which is 0.85 K lower than that of the conventional shape. These results show that the heat dissipation ability of the arc-shaped fins is superior to that of the traditional rectangular fins.

Figure 3c is the temperature field cloud diagram of the rectangular fins. It can be seen from the scale that the maximum temperature on the IGBT module is 318.15 K (=45 °C), and the temperature value gradually increases along the x-axis direction. The average temperature of the IGBT in the second row is 318 K, and the maximum temperature is significantly lower than the other two. And as shown in Figure 4c, the maximum temperature of the chip at the air inlet is 314.87 K, which is also lower than the other two. It can be seen from the entire temperature field, a large amount of heat generated by the chip is transmitted through the fins, leaving only a small amount of heat remaining at the chip. The average temperature at the lower end of the cut surface has dropped to about 306 K, and the lowest temperature is 303.93 K. Compared with the other two, the heat dissipation effect of the rectangular fins is more remarkable. The reason is that the rectangular fin increases the heat dissipation surface area of the radiator compared with the other two, and also increases the air flowability. As a result, the heat dissipation capability can be effectively improved.

**Figure 5:**
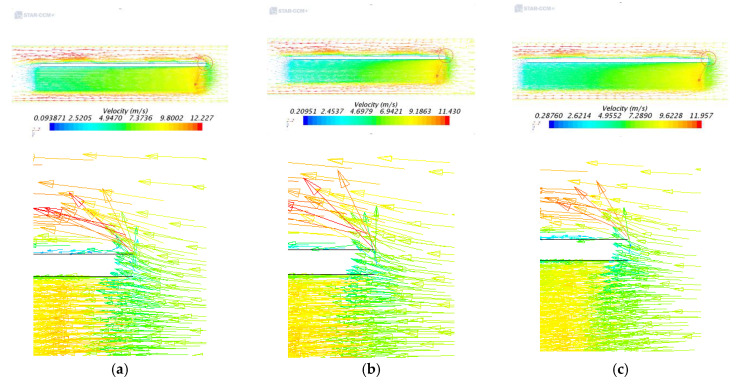
Velocity distributions under different heat dissipation structures. (**a**) Traditional rectangular fins; (**b**) Arc-shaped fins; (**c**) Rectangular fins.

Figure 5 shows velocity distributions. It can be seen from Figure 5a,b that the air flow speed of the upper and lower ends of the IGBT module is the fastest. It can be seen from the three partial enlargement vector diagrams, the front end of the module blocks the airflow at the inlet, so the airflow velocity here is reduced and a small portion of the stagnation region is formed. After that, a part of the airflow flows along the IGBT module to the outlet, and the airflow speed is gradually reduced. When the airflow flows to the rear end of the IGBT module, the speed of the traditional rectangular is reduced to about 3 m/s, while the speed of the arc-shaped is reduced to about 3.5 m/s. It can be seen from Figure 5c, after the airflow is blocked at the front end of the IGBT module, the velocity of the airflow at the rear end of the module is about 4.2 m/s. The reason is that the rectangle increases the fin spacing compared with the other two, improves the flowability of the airflow, which greatly reduces the loss of the airflow speed, and effectively enhances the convective heat transfer capability.

The above results show that all three structures can meet the conventional heat dissipation requirements. The simulation and theoretical calculation results are basically the same. The IGBT junction temperatures under the three structures are theoretically calculated to be 49.35 °C, 48.12 °C, and 47.07 °C, respectively. The IGBT junction temperatures of the three structures obtained by simulation are 46.96 °C, 46.10 °C, and 45 °C, respectively. The error is less than 5%, indicating that the two heat-dissipating fins solutions are feasible. By contrast, it can be seen that the heat dissipation performance of the traditional rectangular fins is lower than the other two, and the heat dissipation performance of the arc-shaped fins is slightly lower than that of the rectangular fins.

### 4.4. Discussion

The factors that influence the performance of the IGBT are discussed here.

**Figure 6:**
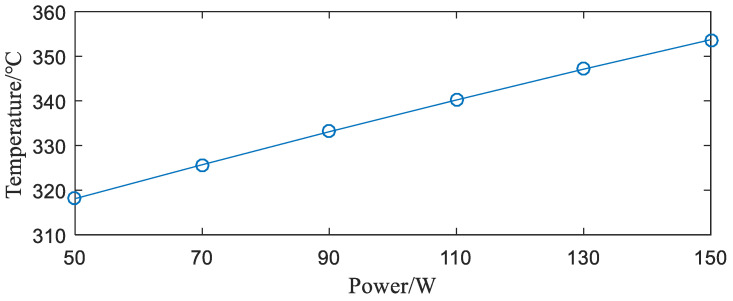
Relationship between power and junction temperature of the IGBT module.

**(1)** Influence of Power. The IGBT module under the rectangular fins was simulated with different power values. The results obtained are shown in Figure 6.

It can be seen from Figure 6 that, for each 20 W increase in power, the maximum temperature change of the module is 7.6 K, 7.4 K, 6.9 K, 7.2 K, and 7.1 K. It shows that when the power starts to increase, the heat dissipation effect of the radiator is obvious. When the power is increased to a certain value, the heat dissipation capability of the radiator will not meet the heat dissipation requirements of the IGBT module, and the module will be burned. In Figure 6, when the power is 150 W, the maximum temperature of the IGBT module is 353.7 K (=80.55 °C), which is still lower than the maximum junction temperature of 125 °C allowed by the IGBT module. The results show that the rectangular fins have strong heat dissipation capability and can meet the normal operation of many IGBT modules. However, this heat sink is not suitable for use with overpowered IGBT modules.

**Figure 7:**
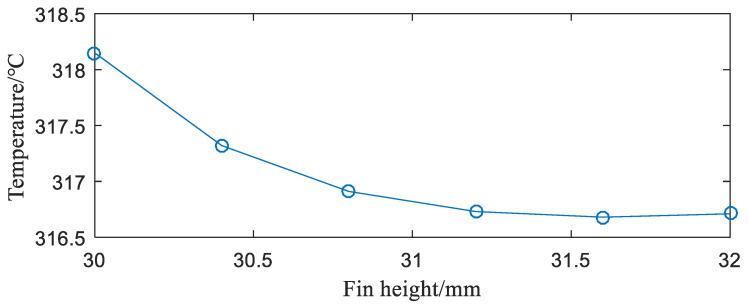
Relationship between fin height and junction temperature of the IGBT module.

**(2)** Influence of fin height. After analyzing the influence of power on the IGBT module temperature, the IGBT module of the rectangular fins was numerically simulated under fins of different heights. The results obtained are shown in Figure 7.

It can be seen from Figure 7 that, as the fin height increases, the junction temperature of the IGBT module decreases. When the fin height is 30.8 mm, the module junction temperature drops significantly. Then, as the fin height increases, the module junction temperature slowly decreases. The reason is that under the same conditions, increasing the fin height is equivalent to increasing the heat exchange area between the radiator and the air, thus the heat dissipation performance is improved. However, when the cost of the material is considered, the fin height should not be too high.

**Figure 8:**
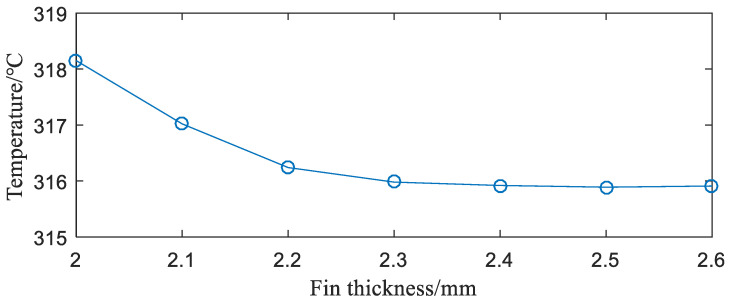
Relationship between fin thickness and junction temperature of the IGBT module.

**(3)** Influence of fin thickness. Fin thickness is one of the important factors affecting the temperature of the IGBT modules. The IGBT module of the rectangular fins as numerically simulated under fin of different thicknesses. The results obtained are shown in Figure 8.

As can be seen from Figure 8, under the same conditions, the greater the thickness of the fin, the better the heat dissipation capability of the IGBT module. However, when the fin thickness is 2.3 mm, the maximum temperature of the IGBT module begins to decrease slowly, and the influence of the fin thickness on the heat dissipation capability of the module begins to decrease. When the fin thickness is 2.5 mm, the maximum temperature of the IGBT module is hardly reduced. At this time, the main factor affecting the heat dissipation performance of the module is convection heat transfer. The influence of fin thickness on it is negligible, so the fin thickness should not be too large.

**Figure 9:**
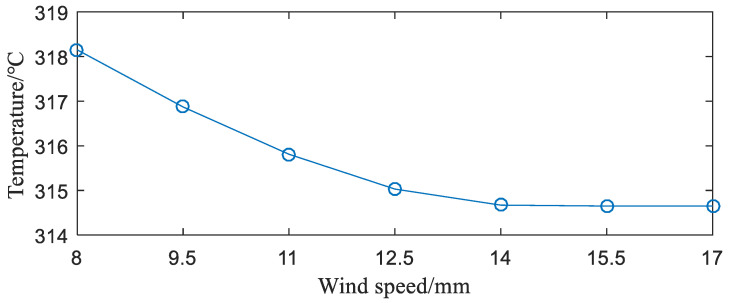
Relationship between wind speed and junction temperature of the IGBT module.

**(4)** Influence of wind speed. For air-cooled radiators, the effect of wind speed on the radiator is critical. The results obtained are shown in Figure 9.

As can be seen from Figure 9, as the wind speed increases, the IGBT module temperature begins to drop. When the wind speed reaches 12.5 m/s, the temperature drop rate of the IGBT module begins to become gentle. As the wind speed continues to increase, the temperature drop has changed little. It is no longer meaningful to continue to increase the wind speed due to factors, such as power consumption and noise. Thus, it is not advisable to increase the wind speed to improve the heat dissipation performance.

## 5. Conclusions

In this paper, two new IGBT module radiators are designed, which are arc-shaped and rectangular radiators. The IGBT module is modeled by the 3D drawing software UG. The IGBT junction temperature is theoretically calculated, and the IGBT module of three different heat-dissipating fins was numerically simulated by STAR-CCM+ software. The results show that the error between the simulation results and the theoretical calculation results are within 5%, which proves the feasibility of the two design schemes. The heat dissipation capability of the arc-shaped and rectangular heat-dissipating fins is superior to that of the conventional heat-dissipating fins, and the rectangular fins are superior to the arc-shaped fins. The effects of fin structure, power, fin height, fin thickness, and wind speed on the heat dissipation performance of the IGBT modules are obtained. The result provides a reference for the structural design and optimization of the finned radiator.

## Figures and Tables

**Table 1:** Material properties.

Material	Density/$\left(kg\cdot m^{-3}\right)$	Thermal Conductivity/$\left(Wm^{-1}K^{-1}\right)$	Specific Heat Capacity/($J\cdot {\left(kg\cdot k\right)}^{-1}$)
Si	2329	124	702
Al	2702	237	903
Cu	8940	398	386

**Table 2:** Temperature field distribution of three structures.

	Temperature Value	A/K	B/K	C/K	D/K	E/K	F/K
Type							
Traditional rectangular		308.17	310.56	312.95	315.34	317.72	320.11
Arc-shaped		307.06	309.50	311.94	314.37	316.81	319.25
Rectangular		305.22	307.80	310.39	312.98	315.56	318.15
